# Anxiety in Chinese Patients With Cleft Lip and/or Palate: A Preliminary Study

**DOI:** 10.3389/fped.2022.842470

**Published:** 2022-02-14

**Authors:** Zhuojun Xie, Chao Yang, Yuxi Zhao, Yichun Yang, Weiyao Xia, Yuan Zong, Ting Chi, Bing Shi, Hanyao Huang, Caixia Gong

**Affiliations:** State Key Laboratory of Oral Diseases and National Clinical Research Center for Oral Diseases and Department of Oral Maxillofacial Surgery, West China Hospital of Stomatology, Sichuan University, Chengdu, China

**Keywords:** cleft lip and/or palate, anxiety, GAD-7, psychology, Chinese CL/P patients

## Abstract

**Objectives:**

To preliminarily analyze factors that affected the prevalence of anxiety in Chinese patients with cleft lip and/or palate (CL/P).

**Methods:**

The Generalized Anxiety Disorder Scale (GAD-7) was used to screen anxiety in Chinese CL/P patients. Non-CL/P individuals were also included as the control group. Sociodemographic and clinical data consisting of diagnosis, gender, only child or not, monthly household income, and current family location were collected to analyze possible factors that could affect the anxiety of this patient population.

**Results:**

One hundred forty-two and 78 valid questionnaires were collected from the study and control groups, respectively. The mean GAD-7 score of the study group (3.092 ± 3.381) was significantly lower than the control (3.987 ± 2.505). Moreover, the proportion of patients presenting with moderate-severe anxiety was larger in the study group than in the control group (6.6 vs. 0.0%). Statistically significant differences in GAD-7 scores were observed between the study and control groups when the patient was the only child, living in an urban area, or the monthly household income was between 1,000 and 5,000 yuan.

**Conclusion:**

Although the severity of anxiety in Chinese CL/P patients was not severer than those without CL/P, there was a relatively high incidence of moderate-severe anxiety in CL/P patients, while the only child, current family location and the monthly household income played significant roles in affecting anxiety psychology.

## Introduction

Cleft lip and palate (CL/P) is one of the most common congenital craniofacial abnormalities, characterized by failure of normal fusion of the palate and lip at the midline during embryonic development, resulting in a clinically obvious deformity of the newborn (1), bringing unignorable appearance malformations and increasing risk for various psychological diseases like loneliness, low self-esteem, social disorders, and mood disorders to patients (2). Although there lies a strong association between psychological wellbeing and appearance, and between psychological wellbeing and social adjustment (3), some other studies demonstrated few differences in psychological status between CL/P patients and those who without appearance malformations (4). It reminds us of the need for further studies.

As a common mental illness, anxiety refers to a feeling of nervousness, impatience, and restlessness about things, accompanied by physical symptoms such as palpitation, chest distress, and hyperventilation (5). It has been reported that compared with individuals without CL/P, CL/P patients are easily faced with relatively higher severity of anxiety (6). As anxiety could affect the life quality of the patients, identifying factors influencing anxiety status in Chinese CL/P patients would provide the theoretical basis for psychological intervention and improve the quality of cleft care. Thus, in this study, we sought to preliminarily investigate the characteristics of anxiety in Chinese CL/P patients by applying the Generalized Anxiety Disorder Scale (GAD-7) to analyze the effect of CL/P on anxiety and the possible related influencing factors.

## Materials and Methods

### Subjects

Chinese Patients with a history of cleft lip and (or) cleft palate who visited West China Hospital of Stomatology, Sichuan University, between July 2019 and January 2021 were enrolled in the study group. Inclusion criteria of the study group were as follows:

- Patients with complete or incomplete unilateral or bilateral non-syndromic cleft lip and (or) palate.
- Patients who underwent cleft lip repair and (or) palatoplasty.
- Patients aged 10 years or above (7).

Through the distribution of questionnaires in the general population, inclusion criteria of the control group were as follows:

(1). Individuals with no significant facial defects or other major diseases.

(2). Individuals aged 10 years or above (7).

Sociodemographic and clinical data were collected for analysis, including diagnosis, gender, only child or not, monthly household income, and current family location.

The research protocol was reviewed and approved by the Ethics Committee of West China Hospital of Stomatology, Sichuan University (No. WCHSIRB-D-2016-084R1). Written informed consents were acquired from all the patients or their parents.

### Measurement of Anxiety Status

The Generalized Anxiety Disorder 7-Item Scale (GAD-7) was used to assess patient anxiety status. Seven items were used to assess the frequency of anxiety symptoms over 2 weeks on a 4-point Likert scale ranging from 0 (never) to 3 (nearly every day). The total score of GAD-7 ranged from 0 to 21, with increasing scores indicating more severe functional impairments due to anxiety. The total score was calculated for each patient and interpreted as follows: normal (0–4), mild (5–9), moderate (10–14), and severe (15–21).

As one of the most widely used assessment scales applied for screening, diagnosis, and severity assessment of anxiety disorders (8), GAD-7 has been transadapted into Chinese culture and validated (9). It can serve as an effective tool for anxiety screening in Chinese adolescents (7).

In present study, all subjects were given questionnaires in Chinese version and guided by trained volunteers to make sure every item was fully understood. Each subject completed the questionnaire independently.

### Statistical Analysis

SPSS 23.0 (IBM Corp., Armonk, NY, USA) is used for statistical analyses, and the count data was represented by mean ± standard deviation (SD). Independent samples-T test was used to compare the two independent variables within groups and the independent variables between groups, while one-way ANOVA was used to compare more-than-two independent variables within groups when the compliance with normal distribution is tested by Shapiro-Wilk. The Kruskal-Wallis test was typically used when the normality assumption was violated. *P*-values < 0.05 were statistically significant, while *P-*values < 0.01 were highly statistically significant.

## Results

### Participant Characteristics

A total of 145 GAD-7 (142 valid and 3 invalid) questionnaires were collected from the study group (Mean age: 17.2 ± 5.8 years), while a total of 78 questionnaires were collected from the control group (Mean age: 18.5 ± 5.2 years). No significant difference in age was found between the two groups. The demographic characteristics of both groups were shown in Table 1.

**Table 1:** The demographic characteristics of the study group and control group.

**Variables**	**The study group**		**The control group**	
	**Number**	**Percentage**	**Number**	**Percentage**
**1. Diagnosis**				
Cleft lip and palate	81	57.0%		
Cleft lip	38	26.8%		
Cleft palate	23	16.2%		
**2. Gender**				
Male	87	61.3%		
Female	55	38.7%		
**3. Only child**				
Only child	45	31.7%	45	57.7%
Non-only child	97	68.3%	33	42.3%
**4. The monthly household income**				
Under 1,000 yuan	27	19.0%	2	2.6%
1,000–5,000 yuan	73	51.4%	27	34.6%
5,000–10,000 yuan	17	12.0%	28	35.9%
More than 10,000 yuan	18	12.7%	21	26.9%
**5. Current family location**				
Rural	73	51.4%	17	21.8%
Urban	69	48.6%	61	78.2%

### Anxiety Status in the Study and Control Groups

The mean GAD-7 scores in the study and control groups were 3.092 and 3.987 points, respectively, and the scores difference was statistically significant (Table 2). Moreover, the proportion of moderate-severe anxiety in the study group was larger than in the control group (6.6 vs. 0.0%) (Table 3).

**Table 2:** The GAD-7 scores of the study group and control group.

	**Mean ±SD**	**Median**	**Range**	**Interquartile Range**	**95% Confidence interval for mean**		***P*-value**
					**Lower bound**	**Upper bound**	
The study group	3.092 ± 3.381	2.000	17.000	3.000	2.531	3.653	
The control group	3.987 ± 2.505	4.000	9.000	4.000	3.423	4.552	
							0.016*

*P < 0.05 by independent samples-T test.

**Table 3:** Severity of anxiety in the study and control groups.

	**Total**	**Normal**		**Mild anxiety**		**Moderate anxiety**		**Severe anxiety**	
		**Number**	**Percentage**	**Number**	**Percentage**	**Number**	**Percentage**	**Number**	**Percentage**
The study group	142	110	77.5%	24	16.9%	6	4.2%	2	1.4%
The control group	78	46	59.0%	32	41.0%	0	0.0%	0	0.0%

### The Analyses of the Factors That Influenced GAD-7 Scores

Sociodemographic and clinical data consisting of diagnosis, gender, only child or not, monthly household income, and current family location did not influence the anxiety status between subgroups in both study and control groups. However, analysis between groups showed a statistically significant difference in subjects that were an only child and lived in an urban location, while a highly statistically significant difference was demonstrated in subjects with a total family income between 1,000 and 5,000 yuan (Table 4).

**Table 4:** Factors affecting anxiety status of Chinese patients with CL/P.

**Variables**	**The study group**		**The control group**		***P*-value (study vs. control)**
	**Mean ±SD**	***P*-value**	**Mean ±SD**	***P*-value**	
**1.Diagnosis**					
Cleft lip and palate	3.123 ± 3.455	0.967			
Cleft lip	2.974 ± 3.234				
Cleft palate	3.174 ± 3.499				
**2.Gender**					
Male	3.046 ± 3.316	0.841			
Female	3.164 ± 3.511				
**3.Only child**					
Non-only child	3.412 ± 3.665	0.081	3.636 ± 2.421	0.292	0.729
Only child	2.326 ± 2.597		4.244 ± 2.560		0.017*
**4.The monthly household income**					
Under 1,000 yuan	4.037 ± 4.808	0.064	4.000 ± 5.659	0.839	0.992
1,000–5,000 yuan	2.493 ± 2.631		4.296 ± 2.826		0.004**
5,000–1,0000 yuan	4.529 ± 3.105		3.964 ± 2.099		0.513
Over 1,0000 yuan	3.167 ± 3.792		3.619 ± 2.439		0.656
**5.Current family location**					
Urban	2.696 ± 3.331	0.181	3.787 ± 2.430	0.183	0.037*
Rural	3.471 ± 3.471		4.706 ± 2.710		0.175

*P < 0.05, ^**^P < 0.01 by independent samples-T test or one-way ANOVA.

## Discussion

It has always been assumed that facial appearance is vital for healthy psychosocial development (10). Given that CL/P patients experience difficulties in feeding, speech dysfunction, and cosmetic defects from birth, their psychological development could be affected (11). However, in contrast, other studies demonstrated no significant difference in psychological status between CL/P patients and non-CL/P individuals (12, 13). In the setting of the fact that research on the incidence of anxiety and its influencing factors of CL/P patients in China were considerably unclear, the present research aimed to gain a deeper insight into the prevalence of anxiety in Chinese CL/P patients and identify potential influencing factors.

Interestingly, we found that the average GAD-7 score of Chinese CL/P patients was lower than that of the control group. Possible explanations were as follows: There were studies that claimed CL/P patients reported better emotional wellbeing and overall self-worth than healthy people, and the impact of CL/P was not worse than having other concerns around appearance (14, 15), given that CL/P reportedly strengthened the individual's vulnerability to disadvantageous psychological experiences when faced with emotional difficulties and negative self-perception, as most of the patients were observed to perform and adapt well when confronted with adversities (16, 17). What's more, as the severity of anxiety could be divided into normal, mild anxiety, moderate anxiety and severe anxiety on the basis of GAD-7 score, we found that the proportion of moderate-severe anxiety in the study group was larger than that in the control group, indicating that CL/P disease might pose a severer negative effect on patient's anxiety. Nonetheless, further studies with larger sample sizes should be conducted to substantiate the potential negative effect exerted by CL/P.

The heterogeneity of cleft types could explain the differences observed in our study. Although the difference was not statistically significant, higher GAD-7 scores were found in patients diagnosed with cleft palate (CP) or cleft lip and palate (CLP) than those with cleft lip (CL), consistent with previous studies. As for CLP patients, they faced more significant challenges in social interactions and communications than other types of CL/P resulting from significant speech difficulties and language delays (18), potentially contributing to the worse psychological status. Additionally, it had been investigated that children with CP showed severer problems with parent- and teacher-reported depression, anxiety, and other psychological difficulties than children with CL or CLP, partially explained by greater speech problems and subsequently poorer learning disorder rather than facial defects because of its morphological abnormities and functional defects.

We also found that female patients scored slightly higher in GAD-7 than males while the difference was not statistically significant, indicating the possibility that female patients experienced relatively worse psychological conditions to some extent. As gender differences have been frequently studied when it came to psychology, it has been demonstrated that females were more likely to experience appearance dissatisfaction (19) and more at risk for emotional distress than males with the same diagnosis due to their higher awareness of sentiment and sensitivity (20).

Moreover, a statistical difference in patients who were an only child was found between CL/P patients and the general. For the sake of reasonable explanation, we found that as parents with children or children with siblings suffering from severe diseases such as cancer were more likely to break the family's normal function and dynamic balance (21), dysfunctional family predicted higher levels of anxiety and other emotional disorders in adolescents, receiving more negative feedback and discouragement of emotional expression (22). However, opposite outcomes have been reported in our study. It's worth noting that even though China's one-child policy ended in 2015, it was in place for over three decades and was mainstream in Chinese families, contributing to the psychosocial differences between groups of only children vs. children with siblings. We found that non-only-children with CL/P scored higher than only-child patients, implying that having siblings might be a risk correlated with anxiety, depression and other psychological disorders (23), partially explained by the so-called resource dilution model (24), meaning that increase in numbers of children bringing about a reduction in available resources from parent to child (25). As for children in general, opposite outcomes have been reported, indicating the complex predictive effects on anxiety in an only child vs. a non-only child group.

In terms of household income, CL/P patients with a monthly household income between 5,000 and 10,000 yuan were documented to have the highest GAD-7 scores, followed by a monthly household income lower than 1,000 yuan. A statistically significant difference in scores was found between groups in subjects with monthly household income between 1,000 and 5,000 yuan, as the control group scored higher than the study group. Given that the general consensus from most published studies was that the severity of anxiety and depression was negatively associated with the household income (26, 27), socioeconomic deprivation could be intricately linked to mental illness. Although low family income implied poor access to healthcare, less educational opportunities, increased risk of unemployment and eventually giving rise to mental illnesses, CL/P patients tend to exhibit stronger resilience in the face of adversity, with enhanced ability to cope and manage stress and negative experiences compared to the general population due to their craniofacial anomaly (17), which might result in a lower degree of anxiety caused by unsatisfied economic conditions. Anyway, a deeper study should be implemented in the future to investigate the specific relationship between economic conditions and CL/P patients' psychological status.

Higher GAD-7 scores were obtained between groups in subjects from rural areas, which could be partly explained by the negative role of their residential location that could influence their self-worth and self-knowledge (28). It is thought that living in undeveloped areas is an essential factor in deciding one's inner awareness and it can cause more psychological distress (29). In our study, a statistical difference in GAD-7 scores was also found between CL/P patients and people without CL/P of the urban area, while the latter scored much higher.

Several limitations need to be noted in our study. The participants of our study were recruited from a limited geographic area and the study was conducted in only one specialized hospital of stomatology in western China. Also, the sample size of our study was not large enough. In addition, there was the outbreak of The 2019 Coronavirus Disease (COVID-19) epidemic during our collection of data, posing a challenge to psychological resilience upon both Chinese society and the international community. Our results might be influenced by the outbreak and prevalence of COVID-19.

## Conclusion

Herein, given the limitations of this study, we preliminarily provided some evidence on the significance of various causes of anxiety in Chinese CL/P patients. Most importantly, we found that the severity of anxiety in Chinese CL/P patients was not severer than those without CL/P.

## Data Availability Statement

The original contributions presented in the study are included in the article/supplementary material, further inquiries can be directed to the corresponding authors.

## Ethics Statement

The studies involving human participants were reviewed and approved by the Ethics Committee of West China Hospital of Stomatology, Sichuan University (No. WCHSIRB-D-2016-084R1). Written informed consent to participate in this study was provided by the participants' legal guardian/next of kin. Written informed consent was obtained from the individual(s), and minor(s)' legal guardian/next of kin, for the publication of any potentially identifiable images or data included in this article.

## Author Contributions

ZX and CY contributed equally to this work. ZX, CY, YZ, YY, WX, and YZ contributed to the collection of data. ZX, CY, YZ, and TC analyzed the data. ZX, CY, BS, HH, and CG contributed to writing and revising the paper. BS, HH, and CG supervised the research. All authors contributed to the article and approved the submitted version.

## Funding

This work was supported by the Research and Develop Program, West China Hospital of Stomatology Sichuan University (RD-02-202107), granted to HH.

## Conflict of Interest

The authors declare that the research was conducted in the absence of any commercial or financial relationships that could be construed as a potential conflict of interest.

## Publisher's Note

All claims expressed in this article are solely those of the authors and do not necessarily represent those of their affiliated organizations, or those of the publisher, the editors and the reviewers. Any product that may be evaluated in this article, or claim that may be made by its manufacturer, is not guaranteed or endorsed by the publisher.
